# Inhibition of NUCKS Facilitates Corneal Recovery Following Alkali Burn

**DOI:** 10.1038/srep41224

**Published:** 2017-01-20

**Authors:** Ming-Wai Poon, Dan Jiang, Peng Qin, Yuelin Zhang, Beiying Qiu, Sumit Chanda, Vinay Tergaonkar, Qing Li, Ian Y. Wong, Zhendong Yu, Hung-Fat Tse, David S. H. Wong, Qizhou Lian

**Affiliations:** 1 Department of Medicine, Li Ka Shing Faculty of Medicine, the University of Hong Kong, Hong Kong SAR, China; 2 Shenzhen Institutes of Research and Innovation, the University of Hong Kong, Hong Kong SAR, China; 3 Department of Ophthalmology, Li Ka Shing Faculty of Medicine, the University of Hong Kong, Hong Kong SAR, China; 4 Institute of Molecular and Cellular Biology, Biopolis, Singapore; 5 Infectious & Inflammatory Disease Center, the Burnham Institute for Medical Research, La Jolla, California, U.S; 6 Central Laboratory, Peking University Shenzhen Hospital, Shenzhen, China

## Abstract

Corneal wound healing involves a complex cascade of cytokine-controlled cellular events, including inflammatory and angiogenesis responses that are regulated by transcriptional chromatin remodeling. Nuclear Ubiquitous Casein and cyclin-dependent Kinase Substrate (NUCKS) is a key chromatin modifier and transcriptional regulator of metabolic signaling. In this study, we investigated the role of NUCKS in corneal wound healing by comparing its effects on corneal alkali burn in *NUCKS* knockout (NKO) and *NUCKS* wild-type (NWT) mice. Our data showed that following alkali-injury, inhibition of *NUCKS* (NKO) accelerated ocular resurfacing and suppressed neovascularization; the cytokine profile of alkali burned corneas in NKO mice showed suppressed expression of inflammation cytokines (IL1A & IL1B); upregulated expression of antiangiogenic factor (Pigment Epithelium-derived Factor; PEDF); and downregulated expression of angiogenic factor (Vascular Endothelial Growth Factor, VEGF); *in vitro*, following LPS-induced NFκB activation, NKO corneal cells showed reduced expression of IL6, IP10 and TNFα. *In vitro,* corneal epithelial cells showed reduced NF-κb activation on silencing of NUCKS and corresponding NFκB-mediated cytokine expression was reduced. Here, we illustrate that inhibition of *NUCKS* played a role in cytokine modulation and facilitated corneal recovery. This reveals a potential new effective strategy for ocular burn treatment.

Corneal wound healing is a process that involves various cellular activities that include inflammation, angiogenesis, migration and proliferation^1^,^2^, and that are regulated by cytokines (IL1A, IL1B, Vascular Endothelial Growth Factor, VEGFA, Pigment Epithelium-derived Factor, PEDF). Fine control of cytokine-mediated cellular events is important to minimise scarring and achieve optimal clinical recovery.

Recently, DNA binding proteins have been described in the feedback loop of the cytokine induced cascade^2^,^3^,^4^,^5^,^6^. A nuclear DNA binding protein, Nuclear Ubiquitous Casein and cyclin-dependent Kinase Substrate (NUCKS), which is widespread in vertebrates and expressed ubiquitously by almost all human cell types^7^, has been reported to be a key chromatin modifier and transcriptional regulator of a number of signaling pathways, including cell death, proliferation and movement^8^. It is also reported to regulate the chronic inflammatory response in metabolic syndrome^9^,^10^ and is thought to be involved in protection of a cell against undesirable factors^11^. Recent research has further suggested a potential role of NUCKS in stress responses leading to selective regulation of gene transcription^12^. These reports show that NUCKS shares many of the important functional properties which are important in modulation of corneal wound healing as well as an ability to precisely regulate the inflammatory response and cytokine release^3^. We therefore aimed to determine whether inhibition of NUCKS would facilitate corneal recovery with a particular focus on its role in cytokine modulation.

We investigated the role of NUCKS in corneal wound healing, focusing on the corresponding inflammatory and angiogenic responses in *NUCKS* knockout (NKO) and *NUCKS* wild-type (NWT) mice following central corneal alkali burn. Our results showed that compared with NWT, NKO mice exhibited faster corneal resurfacing and suppressed angiogenic responses that was associated with fine modulation of cytokines: inflammatory factors (IL1A and IL1B) and angiogenic factor (VEGF) and anti-angiogenic factor (PEDF). Our *in vitro* intracellular data revealed that upon stimulation with lipopolysaccharide (LPS; LPS-induced-NFκB activation), NKO group showed reduced expression on IL6, IP10 and TNFα compared with NWT group. In addition, silencing of NUCKS and stimulation with LPS resulted in reduced NFκB signaling activation and reduced expression of cytokines downstream of the NFκB pathway.

## Results

### Inhibition of NUCKS Accelerates Corneal Resurfacing Following Alkali Injury *in Vivo*

**Figure 1:**
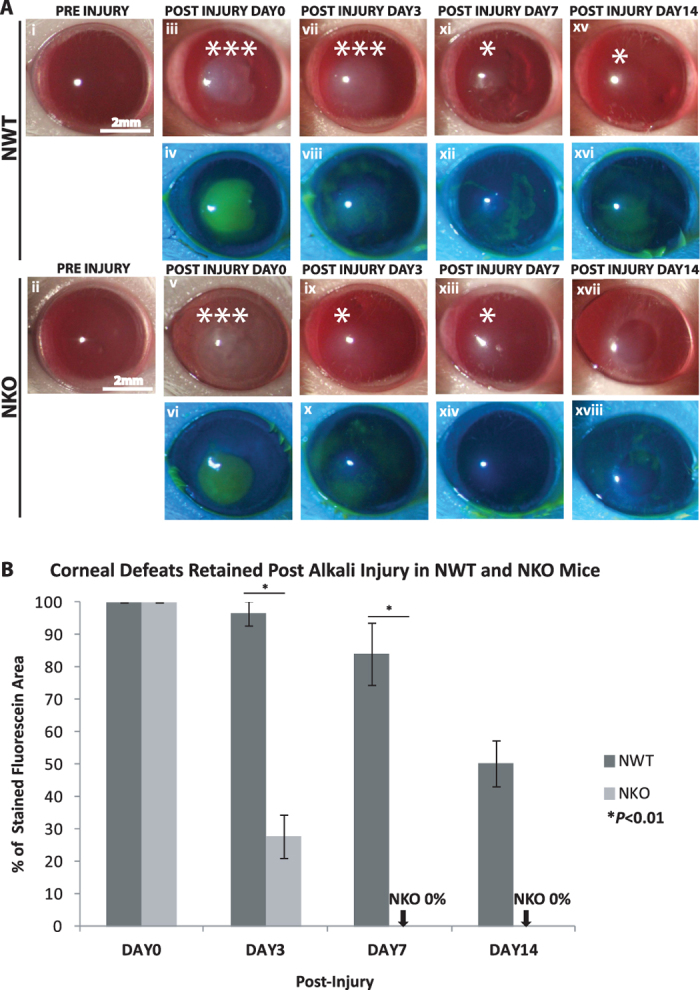
Inhibition of NUCKS Promotes Corneal Resurfacing in Alkali Injured Corneas *in Vivo*. (**A**) Bright field images (upper rows of NWT and NKO columns) and fluorescein images (lower rows of NWT and NKO columns) were captured by slit lamp biomicroscopy. Representative bright field images are shown for NWT (i, iii, vii, xi and xv) and NWT mice and (ii, v, ix, xiii, xvii) at specific time points (pre-injury, days 0, 3, 7 and 14). NKO mice exhibited less severe oedema than NWT mice at the end point, day 14. (NWT*oedema, N = 4; NKO no oedema, N = 4; Scale bars: 2 mm). Representative fluorescein images are shown for NWT (iv, viii, xii and xvi) and NKO mice (vi, x, xiv and xviii). Corneal defeat recovery in NKO mice was significantly faster than that for NWT mice at the end point, post injury day 14. (**B**) Percentage of retained corneal defeat was calculated as follows: the area of defeat retained at a specific time point divided by the area of defeat measured on post injury day 0 (initial point following injury). On day 3, NWT mice showed 96.56 ± 3.89% and NKO mice 27.75 ± 6.61% defeat, indicating that NKO mice achieved better initial healing. As early as day 7, NKO mice showed no defeats 0 ± 0% compared with NWT mice who showed 84.03 ± 9.67% defeat. At the end point on day 14, NWT mice retained 50.18 ± 7.05% defeat and NKO mice exhibited no defeats 0 ± 0%. (*P < 0.01; NWT N = 4; NKO N = 4; Scale bars: 2 mm).

Genotypes of NWT and NKO mice were confirmed as described previously (Supplementary Figure S1)^13^. Alkaline injury was induced within a confined circular area ~2-mm in diameter in the central corneal region in NWT and NKO mice. The effects of inhibition of NUCKS on corneal wound healing following alkali injury were investigated. Bright field and fluorescein images of the corneal healing process were recorded at specific post injury time points (day 0, 3, 7 and 14) (Fig. 1). Using the slit lamp system, a slit beam illumination was generated for the examination of oedema and corneal opacity. Our bright field data showed that faster recovery was observed in the NKO group than the NWT group. On day 14, corneas of the NKO group were transparent whereas those of the NWT group still showed oedema (Fig. 1A, upper panel of NKO; no oedema; upper panel of NWT; ***severe oedema; *oedema). Green fluorescein staining was used in the slit lamp examination, corneal defeat stained green (the defeat at the time immediate after injury, day 0, was defined as 100%) (Fig. 1A). Corneal recovery was notably faster in the NKO group compared with the NWT group. NWT corneas showed positive staining (corneal defeats) on days 3, 7 and 14 (Fig. 1A, lower panel in NWT). In contrast, NKO corneas showed minimal staining on day 3 and no stain was found (stained negative) on day 7 (Fig. 1A, lower panel in NKO). The percentage of retained defeat for NWT and NKO mice respectively was 100 ± 0% and 100 ± 0% on day 0; 96.56 ± 3.89% and 27.75 ± 6.61% on day 3; 84.03 ± 9.67% and 0 ± 0% % on day 7 and 50.18 ± 7.05% vs 0 ± 0% on day 14 (Fig. 1B; *P < 0.01; NKO vs NWT).

### Inhibition of NUCKS Promotes Healing *in Vitro*

**Figure 2:**
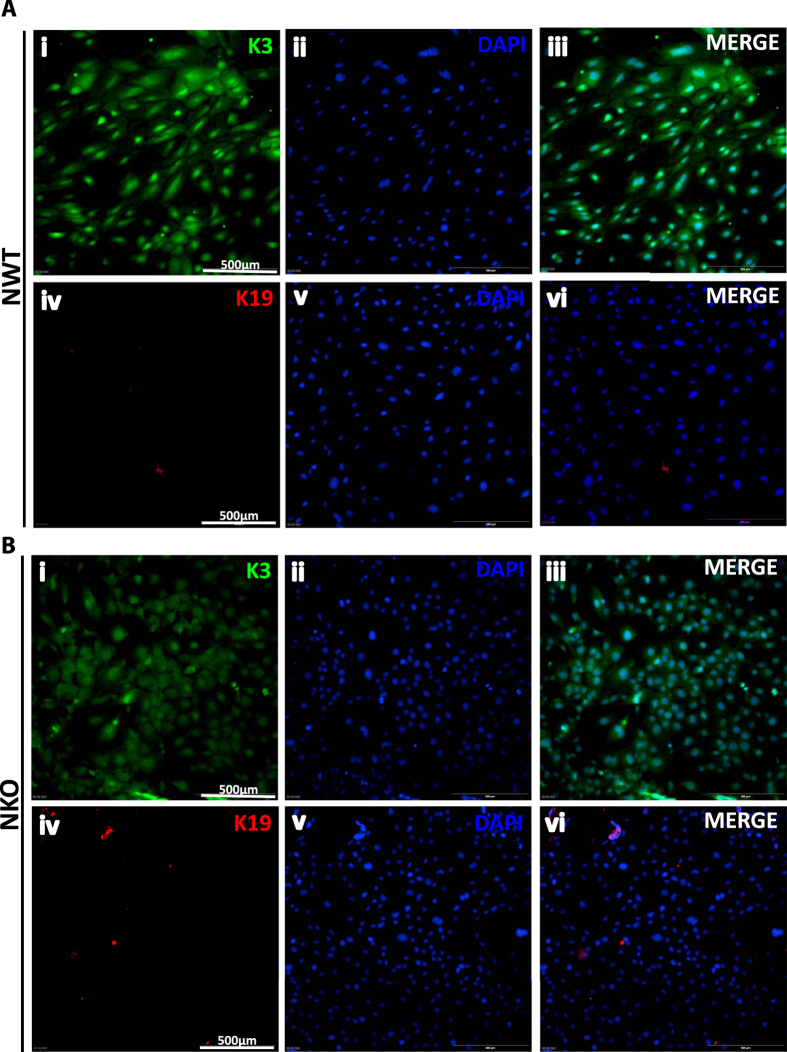
Corneal Epithelial Cells Cultures Isolated from (**A**) NWT and (**B**) NKO mice were Positively Stained with Corneal Epithelial Specific Marker, Cytokeratin K3 on Immunofluorescence Staining. (**A**) Staining for NWT culture. (i) cytokeratin K3 (K3), (ii) DAPI, (iii) combination of K3 with DAPI. (iv) cytokeratin K19 (K19), (v) DAPI, (vi) combination of K19 with DAPI. NWT cell cultures were positively stained with K3 (i, ii, iii), and negatively stained with K19 (iv, v, vi) (NWT, N = 3; NKO, N = 3. (**B**) Staining for NKO culture. (i) K3, (ii) DAPI, (iii) combined K3 and DAPI. (iv) K19, (v) DAPI, (vi) combined K19 with DAPI. NKO mice cell cultures were positively stained with K3 (i, ii, iii) and negatively stained with K19 (iv, v, vi) (NWT, N = 3; NKO, N = 3). Our data confirmed that the isolated cultures from NWT and NKO mice were (~100% K3-positive) pure corneal epithelial cells.

Corneal epithelial marker cytokeratin K3 (K3) and conjunctival epithelial cell marker cytokeratin K19 (K19) were used to confirm the identity of the cultures isolated from the corneas of NWT and NKO groups. Our data showed that both cultures were positively stained for K3 (~100%), but negatively stained for K19 (Fig. 2A and B respectively), indicating that both of the isolated cell cultures were corneal epithelial cells.

**Figure 3:**
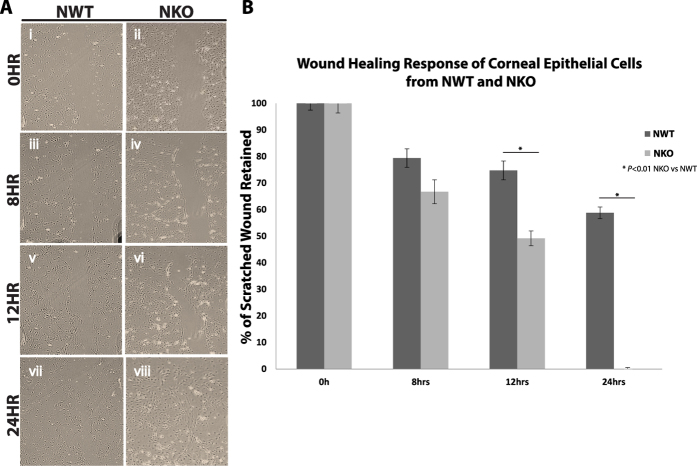
Inhibition of NUCKS Facilities Healing in the Classic Scratched Wound *in Vitro*. Scratched cultures of cells from NWT and NKO mice were monitored for 24 hours and the process was recorded at specific time points (0 hr, 8 hrs, 12 hrs and 24 hrs) under microscopy. **(A)** Representative images were captured and are presented for: (i, iii, v, vii) NWT group and (ii, iv, vi, viii) NKO group at particular time points: 0, 8, 12, and 24 hours. NKO group showed faster wound healing than the NWT group and recovery at 24 hours (NWT, N = 3; NKO, N = 3). Scale bars: 200 μm. **(B)** Graphic representation of the data. NWT group showed 58.82 ± 2.20% retention, while NKO group showed 0.03 ± 0.50% retention after 24 hours. (*P < 0.01; NWT, N = 3; NKO, N = 3).

To examine the effects of inhibition of NUCKS on cell migration, a classic scratch wound healing assay^14^ was performed. By scraping the cultures (NWT and NKO) with a 10 μl pipette tip, a cell-free space was generated (Fig. 3A). Our data demonstrated faster wound closure in the NKO group. The percentage of wound retained for NWT and NKO mice respectively was 100 ± 0% and 100 ± 0% at 0 hour; 79.44% ± 3.5 and 66.75 ± 4.5% at 8 hours; 74.80 ± 3.5% and 49.20 ± 2.8% at 12 hours and 58.82 ± 2.2% and 0.03 ± 0.5% at 24 hours (*P < 0.01; NKO vs NWT).

### Inhibition of NUCKS Reduced the Expression of Inflammatory Cytokines, IL1A &IL1B, Following Alkali Injury *in Vivo*

**Figure 4:**
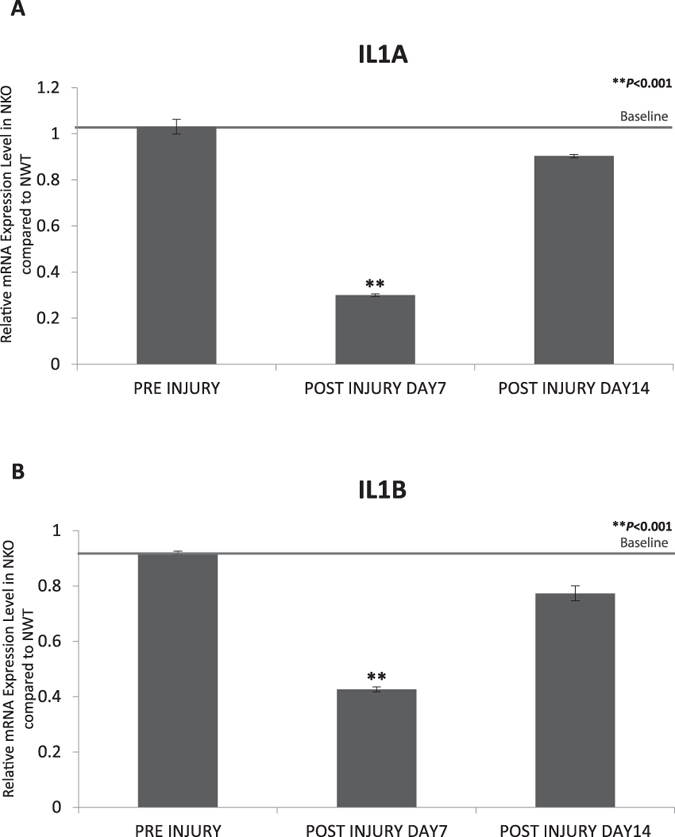
Inhibition of NUCKS Resulted in Reduced Expression of the Key Inflammatory Cytokines, (**A**) IL1A and (**B**) IL1B. Corneas were harvested from NWT and NKO mice at particular time points. The level of expression of IL1A (3.33-fold reduction; **P < 0.001) and IL1B (2.34- fold reduction; **P < 0.001) was reduced in the NKO group on day 7. Level of IL1A and IL1B returned to baseline on by day 14 (NWT N = 12; NKO N = 12).

Following corneal alkali injury, corneas were harvested at various time points from NWT and NKO groups. Since IL1 is a master gene that regulates inflammation and other diverse cellular responses in corneal recovery^2^ and the expression of IL1A and IL1B peaks at day 7 post alkali injury^15^,^16^, we chose to study IL1A and IL1B at day 7. Our data also showed full recovery of NKO corneas by day 14 that was consequently taken as the end-point in our cytokine study. The relative level of expression of NKO was calculated as follows: all the expression levels in NWT and NKO mice were normalized to the corresponding expression levels of GAPDH. The relative level of expression at a particular time point for NKO mice was taken as the ratio of expression in NKO mice compared with that in NWT mice at that particular time point. The baseline level pre-injury, which was around 1, indicated that expression in NKO mice was similar to that of NWT mice at the initial time point (normal condition without injury); on the contrary at subsequent time points a lower relative expression than baseline in NKO mice indicated downregulation of cytokine expression in NKO, and vice versa. Our data showed that in NKO mice. the expression of IL1A and IL1B was reduced (reduced by 3.33 fold and 2.34 fold respectively for IL1A and IL1B; **P < 0.001) on day 7 compared to NWT mice. On day 14, the expression level of NKO for IL1A and IL1B returned to baseline level (Fig. 4A and B). Taken together, inhibition of NUCKS is associated with precise regulated expression of inflammatory cytokines, IL1A and IL1B, following injury.

### Inhibition of NUCKS Reduced Angiogenic Response Following Alkali Injury *in Vivo*

**Figure 5:**
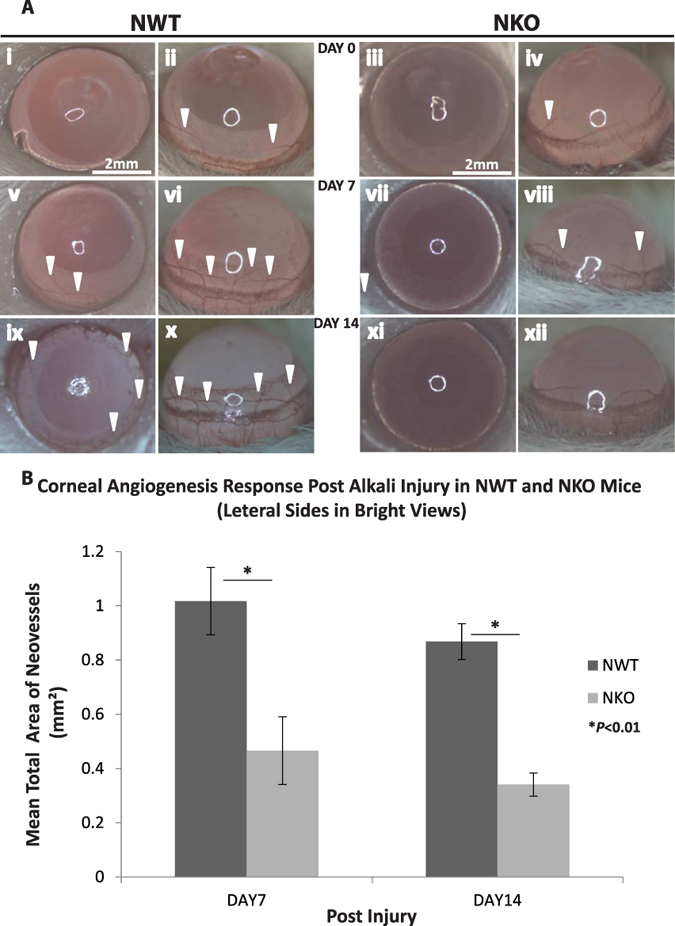
Inhibition of NUCKS Resulted in a Reduced Angiogenic Response *in Vivo*. (**A**) Representative (dorsal and lateral) corneal neovascularization photographs at specific time points (post injury day 0, 7, and 14) of (i, ii, v, vi, ix and x) NWT and (iii, iv, vii, viii, xi and xii) NKO groups are shown. NKO group showed less severe angiogenic response compared with NWT group on day 7 and day 14 (neovascularization marked by arrows; Scale bars: 2 mm). (**B**) Graphic representation of the mean total area of neovascularization. ImageJ was used for the analysis. Compared with NWT, NKO mice exhibited a notable reduction in angiogenic response on day 7 (NWT and NKO; 1.017 ± 0.124 mm^2^ and 0.466 ± 0.125 mm^2^; *P < 0.01) and day 14 (NWT and NKO; 0.868 ± 0.066 mm^2^ and 0.341 ± 0.043 mm^2^; *P < 0.01) (NWT, N = 4; NKO, N = 4).

The effect of NUCKS deletion on corneal neovascularization *in vivo* was analyzed. Alkali was administered to the central region of the corneas in NWT and NKO mice and the neovascularization process monitored for 14 days (at the time point: post injury days 0, 7, and 14). The total area of blood vessels was recorded and analyzed with software ‘Image Processing and Analysis in Java’ (Image J; Wayne Rasband, National Institute of Mental Health, Bethesda, Maryland, USA) for both the NWT and NKO groups (Fig. 5A). Our data showed that a reduced corneal angiogenic response was observed in the NKO group compared with the NWT group. In the NWT group, a greater response of corneal angiogenesis was observed on days 7 to 14; on the contrary in the NKO group corneal angiogenesis was reduced on day 7 and gradually diminished on day 14 (Fig. 5A). The mean area of corneal neovascularization for NWT and NKO mice was 1.017 ± 0.124 mm^2^ and 0.466 ± 0.125 mm^2^ respectively on day 7; and 0.868 ± 0.066 mm^2^ and 0.341 ± 0.043 mm^2^ on day 14 (*P < 0.01; Fig. 5B).

### Inhibition of NUCKS Downregulated Expression of Angiogenic VEGF and Upregulated Expression of Anti-Angiogenic Factors (PEDF) Following Alkali Injury *in Vivo*

**Figure 6:**
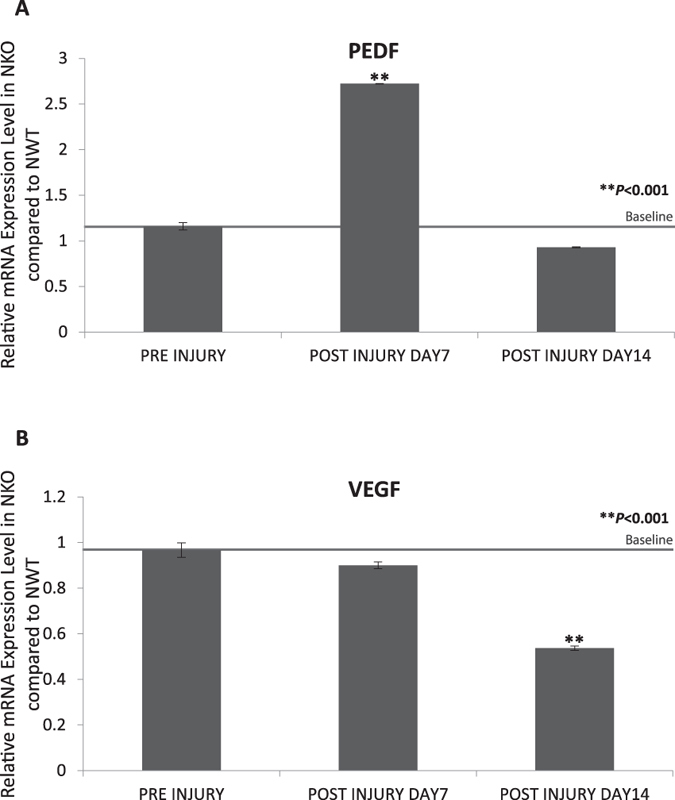
Inhibition of NUCKS Resulted in Reduced Expression of Antiangiogenic and Angiogenic Cytokines, (**A**) PEDF and (**B**) VEGF. Following injury corneas were harvested from NWT and NKO mice at particular time points. Compared with NWT mice, the relative expression level of PEDF (~2.63-fold, **P < 0.001) was upregulated in NKO mice on day 7 and returned to normal on day 14. Furthermore, the relative expression level of VEGF was downregulated (~1.86-fold, **P < 0.001) in NKO mice on day 14 (NWT, N = 12; NKO, N = 12).

We investigated the role of NKO in the angiogenic response of the cornea following alkali-burn by comparing the particular responses in NKO and NWT mice. Corneas were harvested at particular time points following injury, and RNA extracted, converted for cDNA and analyzed using real time RT-PCR. The expression of anti-angiogenic and angiogenic cytokines, PEDF and VEGF, respectively, was examined. As for our previous data in Fig. 1, NKO mice showed full recovery on day 14, thus day 14 was set as the end point. The relative level of expression by NKO mice was calculated as follows: all expression levels of NWT and NKO mice were normalized to the corresponding expression level of GAPDH. The relative level of expression at a particular time point for NKO mice was taken as the ratio of expression of NKO mice compared with that of NWT mice at that particular time point. The baseline level pre-injury, which was around 1, indicated that expression by NKO mice was similar to that of NWT mice at the initial time point (normal condition without injury); at subsequent time points, there was a lower relative expression by NKO mice compared with baseline indicating downregulation of cytokine expression. In NKO mice, mRNA expression of PEDF was upregulated (2.63 fold; **P < 0.001) in corneas on day 7, and returned to the basal level on day 14 (Fig. 6A) compared to NWT mice. In addition, the level of expression of VEGF remained the same as baseline on day 7 and was downregulated on day 14 (1.86 fold; **P < 0.001) in NKO mice corneas (Fig. 6B) compared to NWT mice. Our data demonstrate that inhibition of NUCKS precisely regulated the expression level of antiangiogenic and angiogenic factors to enable good recovery of injured corneas.

### NUCKS regulates NF-κB Activation

**Figure 7:**
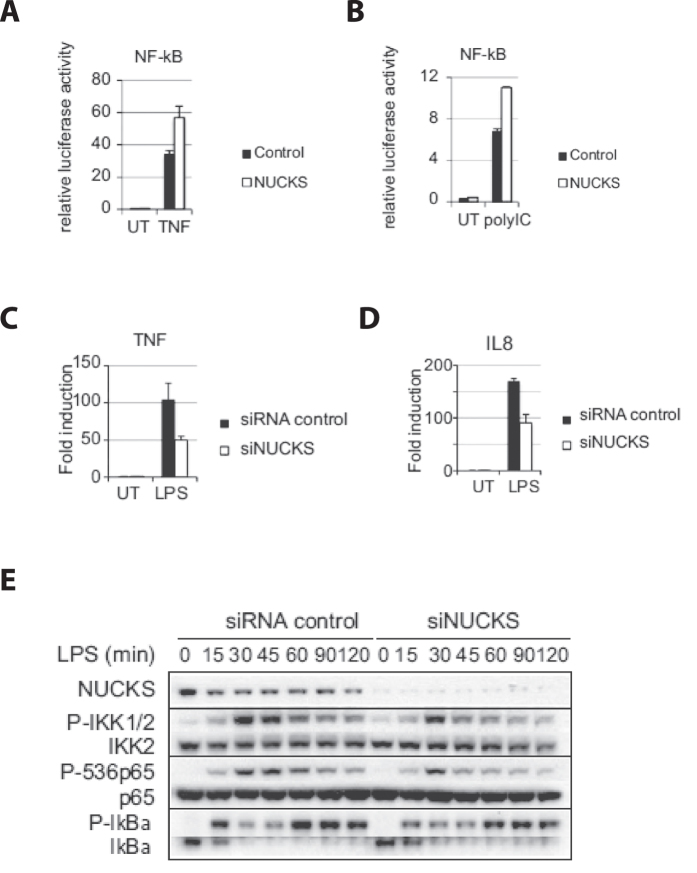
NUCKS regulates NFκB Activation in *in Vitro* Studies. Cells were transfected with luciferase reporter and the corresponding luciferase activity represented intracellular NFκB/ISRE activity. Next, the NUCKS expressing construct (NUCKS) or empty vector (Control) was transfected to the cells, followed by (**A**) TNFα treatment or no treatment (UT); NFκB activation was measured (**B**) lipopolysaccharide (LPS) or no treatment (UT); (i) NFκB activation was measured and (ii) interferon stimulated response element (ISRE) activation was measured. **(C)** Poly-inosinic-cytidylic (polyIC) treatment or no treatment (UT); (i) NFκB activation was measured and (ii) interferon stimulated response element (ISRE) activation was measured. (**D**) On NUCKS silencing and LPS-induced-NFκB activation, the level of expression of (i) TNFα, (ii) IL8 and (iii) MCP1 was tested. (**E**) On NUCKS silencing and LPS-induced-NFκB activation, protein expression level of the phosphorylated form of IKK, P65 and IκBα was analyzed.

The relationship between NFκB and NUCKS was examined *in vitro*. Corneal epithelial cells were transfected with luciferase construct that reflects the cellular activities of the corresponding signaling pathways. NUCKS-overexpressed cells, compared with null-overexpressed cells (control), showed enhanced NFκB activities upon TNF activation (Fig. 7A). Similarly, overexpression of NUCKS also enhanced lipopolysaccharide (LPS)-induced NFκB activation (Fig. 7Bi) and LPS-induced-interferon-stimulated response element (ISRE) activation (Fig. 7Bii). Upon Poly-inosinic-cytidylic (polyIC) treatment, NFκB activation (Fig. 7Ci) and ISRE activation (Fig. 7Cii) were also enhanced by overexpressing NUCKS. On the contrary, we demonstrated that silencing of NUCKS along with the stimulation of LPS reduced expression of TNFα (Fig. 7Di), IL8 (Fig. 7Dii) and MCP1 (Fig. 7Diii). We also found that upon silencing of NUCKS and LPS stimulation, the phosphorylation of IKK, p65 proteins and IκBα (PIκB; a key for the activation NFκB cascade) was impaired 45 mins post- LPS stimulation until the end point of 120 minutes (Fig. 7E). With the above data, we demonstrated that NUCKS regulated NFκB and ISRE activation, the expression of the particular NFκB-mediated cytokines and the phosphorylation of particular proteins critical for the signaling pathway in ocular epithelial cells.

### LPS-induced-NFκB-Activation of NKO Corneal Epithelial Cells Showed Reduced Expression of Phosphorylated IκB (P IκB), IL6, IP10 and TNFα

**Figure 8:**
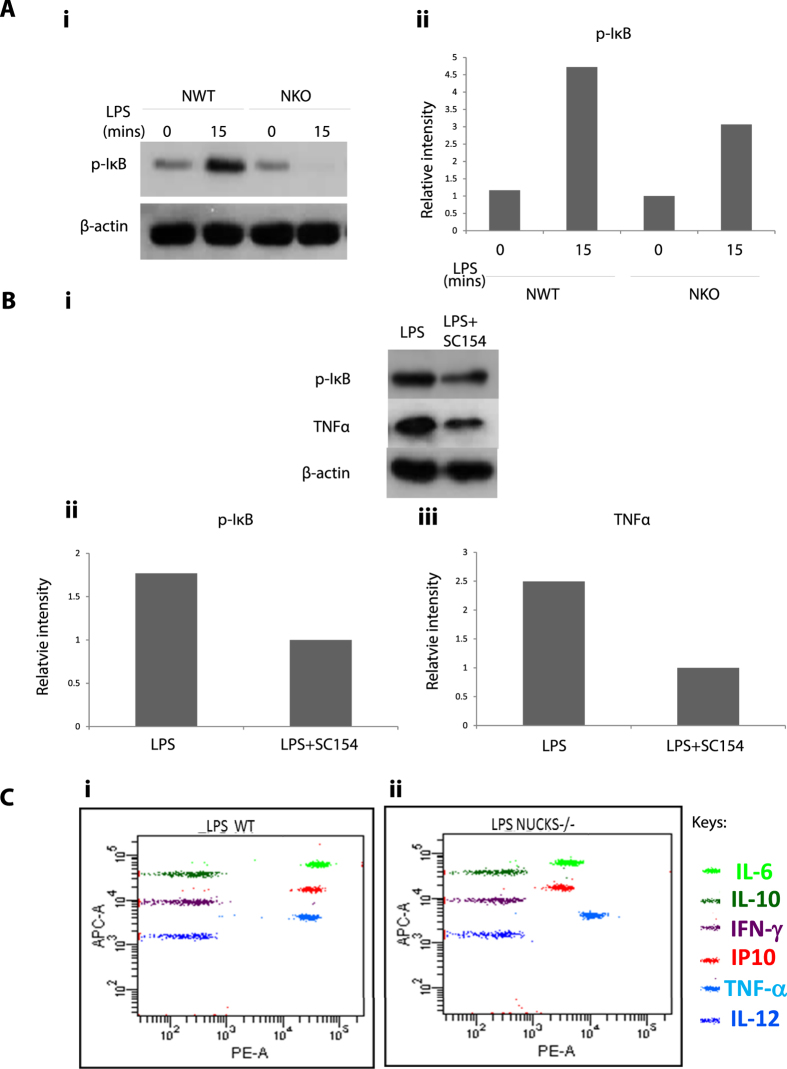
NKO Corneal Epithelial Cells upon LPS-induced-NFκB-Activation Showed Reduced Expression of Phosphorylated IκB (P IκB), IL6, IP10 and TNFα Compared to NWT Group. (**A**) (i) Corneal epithelial cells isolated from NWT and NKO mice were treated with LPS *in vitro*; expression of PIκB was measured (ii) Graphic representation for PIκB data in (i) is shown. (**B**) (i) Corneal epithelial cells were treated with LPS or LPS + NFκB inhibitor, SC 154. Expression of PIκB and TNFα was tested. (ii and iii) Graphic representation of the relative expression of (ii) PIκB and (iii) TNFα, respectively in (i) is shown. (**C**) On treatment with LPS, expression of various inflammatory cytokines (IL6, IL10, INFγ, IP10, TNFα and IL12) was compared and analyzed by flow cytometry analysis. Results for (i) NWT group and (ii) NKO group are shown.

We further tested the effects of inhibition of NUCKS and LPS-induced NFκB activation on corneal epithelial cells isolated from NWT and NKO mice *in vitro*. The expression of phosphorylated IκB and various inflammatory cytokines was investigated. Upon LPS induction, NKO mice showed reduced expression of PIκB compared with NWT mice. To confirm the validity of the results shown in Fig. 8Ai and ii, a control experiment was performed with the NFκB inhibitor, SC154, and corneal epithelial cells *in vitro*. Expression of PIκB and TNFα (Fig. 8Bi–iii) was tested on LPS-treated or LPS + SC154 treated corneal epithelial cells. The NKO mice showed similar effects with the NFκB inhibitor, SC-154, demonstrating reduced expression of PIκB and TNFα. Next, we investigated the effects of inhibition of NKO on expression of inflammatory cytokines (IL-6, IL-10, INFγ, IP10, TNFα and IL-12), upon LPS-induced NFκB activation. The NKO group showed reduced expression of various pro-inflammatory cytokines (IL6^17^, IP10^18^ and TNFα^19^) compared with the NWT group (Fig. 8Ci and ii). (Full-length western blot images are presented in Supplementary Figure S2).

## Discussion

Cellular events, such as cell movement, apoptosis and cell proliferation, followed by scar formation are critical to the corneal recovery process^1^,^2^. Each specific cellular check-point in the recovery process is precisely maintained by a balance of the specific cytokines that are expressed in a timely manner and facilitate the delicate process of corneal healing^2^. Among the various pathways involved in controlling cytokine regulation, the NFκB signaling pathway is widely recognized and studied. NFκB is a principal transcription factor that controls various biological processes, including corneal wound healing, inflammation^20^, angiogenesis, apoptosis and the stress response^21^,^22^,^23^,^24^,^25^. NFκB is also thought to be involved in several ocular surface disorders, including chemical injury, microbial infections, dry eye, pterygium, ultraviolet radiation-induced injury and corneal graft rejection^26^. We have reported previously that a regulator of NFκB, RAP1^4^,^27^, plays a role in angiogenesis and the inflammatory response^28^ and corneal recovery is enhanced via deletion of *RAP1*. Recently, genome-wide ChIP sequencing proved that NUCKS could bind more than 1000 genes involved in inflammation^12^. It is possible that NUCKS, as a transcription factor, is involved in the regulation of NFκB activation, the release of NFκB-mediated-cytokine and the particular cellular events driven by these particular cytokines for inflammation and angiogenesis, thus resulting in an optimal clinical recovery of burnt corneas^29^,^30^.

Our data demonstrated *in vivo* that deletion of *NUCKS* facilitated corneal resurfacing and reduced angiogenic responses following alkali burn. Furthermore, we showed that inhibition of *NUCKS in vivo* modulated inflammatory and angiogenic cytokines during the wound healing process. On LPS-induced NFκB activation of isolated corneal epithelial cells from NWT and NKO mice, NKO mice showed reduced expression of PIκB and inflammatory cytokines *in vitro* compared with the NWT group. Upon either stimulation of TNF, LPS or polyIC, overexpression of *NUCKS* enhanced NFκB and ISRE activation, whereas silencing of *NUCKS* reduced expression of NFκB- mediated cytokines (TNF, IL8, MCP1) and reduced the phosphorylation of IKK2, P65 and IκBα *in vitro* in corneal epithelial cells. Others have reported that *NUCKS* is a key chromatin modifier and transcriptional regulator of a number of signaling pathways and is involved in regulation of chronic inflammation in metabolic syndrome^9^,^10^. *NUCKS* also plays a role in cell protection against undesirable factors^31^ and cell proliferation, apoptosis and cell movement^8^. In this study, our data provided further evidence that *NUCKS* enhanced NFκB activation, and modulated the expression of NFκB-mediated cytokines, regulated NFκB-mediated phosphorylation of IKK2, P65 and IκBα and modulated the various specific inflammatory and angiogenic cytokines.

In our model of alkali injury in NKO mice, expression of the inflammatory cytokines, IL1A and IL1B was downregulated. IL1A is the master gene in wound healing and released first for epithelialization and in response to inflammation^1^,^32^. Meanwhile IL1B plays a role in various cellular events such as fibrosis, cell proliferation, apoptosis, and differentiation^33^,^34^. Our data on the downregulation of these inflammatory cytokines, IL1A and IL1B, were shown to follow the fine healing process *in vivo.* Similar mechanisms of precise regulation of inflammatory cytokines in good recovery have also been reported previously^35^,^36^.

Angiogenesis is another crucial factor during the healing process. The fine balance of angiogenic and anti-angiogenic cytokines (eg. VEGF and PEDF) is important for the survival, apoptosis of corneal endothelial cells^2^,^5^,^6^. In our study, PEDF was highly upregulated on day 7 and returned to baseline on day 14; expression of VEGF was repressed on day 14. A synchronized expression pattern was demonstrated during the healing process in the NKO group *in vivo*. Our data demonstrated a precise regulation of the expression of specific inflammatory and angiogenic cytokines in the corneal recovery process following inhibition of *NUCKS.* This is consistent with our previous report on RAP1, a NFκB regulator, that controls cytokine homeostasis and that is critical for fine control of various cellular events in corneal healing^28^. Here, our data showed a similar mechanism of cytokine-modulated recovery by controlled activation of NFκB, similar to previous findings^4^,^28^,^35^,^36^.

In summary, this is a first study to describe the role of *NUCKS* in corneal wound healing, inflammation and angiogenesis and links *NUCKS* to the activation of NF-κB and modulation of various cytokines. Our findings shed light on *NUCKS* (silencing of *NUCKS*) as a new therapeutic target for fine recovery of the injured cornea.

## Materials and Methods

### The Transgenic Mouse Lines NWT and NKO

All animal experiments were performed in accordance with relevant guidelines and regulations by the University of Hong Kong and approved by the Committee on the Use of Live Animals in Teaching and Research’ (CULATR) (Approval ID: 2533–11). The transgenic mouse lines (NWT and NKO) were gifts from Dr Vinay Tergaonkar (IMCB), the Laboratory of NFκB Signaling, Singapore^13^. Genotypes of all NWT and NKO mice were confirmed (full-length gel image is presented in Supplementary Figure S1) (primary antibody: anti-NUCKS antibody; Abcam 1:200; corresponding secondary antibody: Cell Signaling, 1:1000).

### Induction of Central Corneal Alkali Injury on Corneas of NWT and NKO Mice (in *Vivo*)

After general anaesthesia, 3 μL of 1 N sodium hydroxide solution was applied to the corneas (confined to a circular area ~2-mm in diameter at the central corneal region) of adult NWT (N = 41) and NKO (N = 41) mice^28^.

### Corneal Resurfacing Following Alkali Injury in NWT and NKO Mice (*in Vivo*)

1. Examination of the degree of oedema and opacity on corneal healing was performed using a slit lamp (Catalogue number: KJ5D II, LANLING, KangJie Medical Co., Ltd.). Bright view images were recorded at specific time points (pre injury, post injury days 0, 3, 7 and 14).
2. Investigation of the process of corneal resurfacing on corneal healing was performed with fluorescein stain (Fluorescein sodium ophthalmic strips, register number: HK49096, Contacare Ophthalmics and Diagnostics, Mfg. Lic. No. G/1197) and a slit lamp system. Fluorescein-stained corneal surface defeats were green upon excitation. Fluorescein images were recorded at particular time points (pre injury, post injury days 0, 3, 7 and 14) (NWT, N = 4; N; NKO, N = 4).
3. Investigation of the molecular role of NUCKS in the inflammatory response was performed with real time RT-PCR on isolated RNA from corneas of NWT and NKO mice at particular time points (before injury, days 7 and 14; NWT, N = 12; N; NKO, N = 12). Key inflammatory cytokines IL1A and ILB were studied.

### Corneal Angiogenic Response Following Alkali Injury in NWT and NKO Mice (*in Vivo*)

1. To investigate the degree of angiogenic response in corneal healing, bright view microphotography was performed (NikonSM2800) (Chinetek Scientific Cat No. Infinity 1–3C)^28^. Bright view images were recorded at specific time points (days 0, 7 and 14) (NWT, N = 4; N; NKO, N = 4);
2. To understand the molecular role of *NUCKS* in the angiogenic response *in vivo*, corneas were harvested at particular time points (baseline, post-injury days 7 and 14; NWT, N = 12; N; NKO, N = 12) and total RNA extracted and analyzed with real-time RT-PCR for key angiogenic (VEGF) and anti-angiogenic (PEDF) cytokines.

### Total RNA Extraction, Reverse Transcriptase Reaction (RT-reaction) and Real-time Reverse Transcriptase -Polymerase Chain Reactions (real-time RT-PCR)

Total RNA was extracted using a traditional method by trizol (RNAiso Plus (Cat. No. H9108B, TaKaRa) and converted to cDNA (Cat. No. RR047A, TaKaRa). Real-time RT PCR was performed with CYBR green and a real-time RT-PCR system (Cat. No. HRR820 A, TaKaRa; StepOnePlus™ Real-Time PCR Systems; Thermofisher) according to the manufacturer’s instructions. Specific primers (Supplementary Table 1) were used for the analysis^28^ (NWT; N = 24; NKO; N = 24).

### Primary Cultures of Corneal Epithelial Cells Isolated from NWT and NKO Mice for *in Vitro* Study

The limbus region on the cornea was retained for the growth of epithelial cells. After harvesting, the corneas were cut into 1 mm^2^ sections and cultured on a thin film of SHEM medium [SHEM formulation: DMEM/F12 (Cat. No. Hyclone SH30023.01) FBS (Cat. No. 16000–044, Life Technologies) 5% HEPES (Cat. No. 151630–080, GIBCO) Insulin-Transferrin-Selenium (ITS; 100×) (1%) (Cat. No. 51500–056, GIBCO); (100×) Hydrocortisone (0.5 μg/ml) (Cat. No. 0025012ID, Life Technologies); Dimethyl sulfoxide (DMSO; 0.5%) (Cat. No. 67685, Sigma); and EGF (2 ng/ml) (Cat. No. PHG0311, Life Technologies)]. After 24–72 hrs, a monolayer of corneal epithelial cells would spread out from the sections. SHEM medium was replaced with Keratinocyte Serum-Free Medium (KSFM; Cat. No. 17005–042, Life Technologies; formation as described in manufacturer’s protocol). SHEM and KSFM was applied on alternate days to the cultures, and a monolayer culture was maintained for downstream assays^28^. (NWT, N = 3; NKO, N = 3).

### The Identity of Corneal Epithelial Cells of the Isolated Cultures from NWT and NKO Was Confirmed by Immunofluorescence

Immunofluorescence staining using cytokeratin K3 (a specific marker of corneal epithelial cells; Santa Cruz Cat No. SC-49179; 1:200) and cytokeratin K19 (a specific marker of conjunctival epithelial cells; Santa Cruz Cat No. Sc56371; 1:200) together with the corresponding secondary antibodies (Cell Signaling Technology; 1:1000) was performed to identify the isolated cell cultures from NWT and NKO mice^28^. A LEICA DMI6000B microscope (Leica Microsystems GmbH) was used for imaging (NWT, N = 3; N; NKO, N = 3).

### Classic Wound Healing Scratch Assay *in Vitro*

Classic wound healing scratch assay^14^,^28^,^37^ was performed by scraping the culture with a sterile 10 μl pipette tip (Tip One, USA, Scientific Ocala, FL) on a confluent monolayer culture. Wound recovery was recorded at particular time points (0, 8, 12 and 24 hrs) with a light microscope (Nikon Eclipse TS100-F, Nikon, Japan.) and images were captured using a digital Camera (Nikon Digital Camera D70S, Nikon, Japan.). Scratch assay was performed on NWT and NKO corneal epithelial cultures (NWT, N = 3; N; NKO, N = 3).

### Role of NUCKS in NFκB Activity and Modulation of Cytokines *in Vitro*

*NUCKS* (1 ug; Promega) pISRE-Luc (0.3 μg; Strategene) and Renilla luciferase construct (0.2 μg; Promega) were transfected with Lipofectamine 2000 (Invitrogen) and siRNA(50 nM; Dharmacon and Qiagen) with RNAiMax (Invitrogen). Cells were stimulated with the indicated ligands 24 hours post-transfection and assayed for luciferase activity at particular time points following stimulation by Dual-luciferase reagent (Promega) according to the manufacturer’s instructions.

### Flow Cytometry Analysis of the Expression of Cytokines in Corneal Epithelial Cells Isolated from NWT and NKO Mice *in Vitro*

After 2 hrs of incubation with LPS, corneal epithelial cells were harvested and analyzed with flow cytometry to determine the expression of various cytokines using PE- conjugated or APC- conjugated specific antibodies against IL6, IL10,IP10, INFγ, TNFα and IL12 (BD Biosciences. San Diego, CA), and isotype-matched immunoglobulins (as negative controls; BD Biosciences). BD FACSCalibur flow cytometry (BD Biosciences, NJ) was performed in accordance with the manufacturer’s instructions. (N = 5). Briefly, corneal epithelial cells in culture were detached using Accutase^®^ Solution (Sigma Cat. No. A6964), then washed with sorting buffer (PBS with 1% FBS/BSA and 2 mM EDTA, keep 4 °C). Cells (10^6^–10^7^) were fixed, washed, resuspended again in 100 ul sorting buffer and incubated for 15 mins at room temperature with specific antibodies. Cells were then washed twice with sorting buffer and suspended (cell density = 10^6^ cells/ml) for flow cytometry analysis^38^.

### Western Blotting Analysis of Expression of PIκb and TNFα

To investigate the response of LPS-induced activation of NFκB in corneal epithelial cells; we incubated corneal cells (NWT and NKO) with LPS for 0 and 15 mins. Cells were then harvested for Western Blotting analysis^39^ using specific antibodies for PIκb (abcam). The housekeeping gene β-actin was used for normalization (abcam).

To investigate the response of LPS-induced activation of NFκB to treatment with a NFκB inhibitor SC-154, NWT mice corneal cells were incubated with (i) LPS or (ii) LPS combined SC-154, respectively for 15 mins and cells were harvested for Western Blotting analysis. Specific antibodies for PIκb and TNFα (1:1000 abcam) were used. The housekeeping gene β-actin was used for normalization (abcam). Specific secondary antibodies (1:1000; Dako, USA)^39^,^40^ were used. Intensity of the bands was quantified using the Image Processing and Analysis in Java (Image J) software (Wayne Rasband.Research Services Branch, National Institute of Mental Health, Bethesda, Maryland, USA).

### Statistical Analysis

The mean value of the samples belonging to either NWT or NKO groups are presented ± SD; unpaired student’s t-test was used to determine whether a difference existed between two groups. *P*-values ≤ 0.05 were considered statistically significant. The tests were two-tailed. Standard deviation (±s.d.) was applied for the error bars.

## Additional Information

**How to cite this article:** Poon, M.-W. *et al*. Inhibition of NUCKS Facilitates Corneal Recovery Following Alkali Burn. *Sci. Rep.* **7**, 41224; doi: 10.1038/srep41224 (2017).

**Publisher's note:** Springer Nature remains neutral with regard to jurisdictional claims in published maps and institutional affiliations.

## Supplementary Material

Supplementary Information
