# Supplementary material for: Inhibition of NUCKS Facilitates Corneal Recovery Following Alkali Burn
Source: Sci Rep. 2017 Jan 20;7:41224. doi: 10.1038/srep41224 (PMC5247723; doi:10.1038/srep41224)
Supplement: Supplementary Information [file srep41224-s1.pdf]

Inhibition of NUCKS Facilitates Corneal Recovery Following Alkali Burn

Ming-Wai Poon [1-3], Dan Jiang[3], Peng Qin[3], Yuelin Zhang[3], Beiying Qiu[4], Sumit Chanda[5], Vinay Tergaonkar[4], Qing Li[3], Ian Y. Wong[3], Zhendong Yu[6], Hung-Fat Tse [1], David SH Wong[3], and *Qizhou Lian [1-3*].

(Supplementary Information)

This file contains

1. **Supplementary Figure S1**
2. **Supplementary Figure S2**
3. **Supplementary Table 1**
4. **Figure Legend for Supplementary Figure S1**
5. **Figure Legend for Supplementary Figure S2 and**
6. **Figure Legend for Supplementary Table 1.**

Supplementary Figure- S1

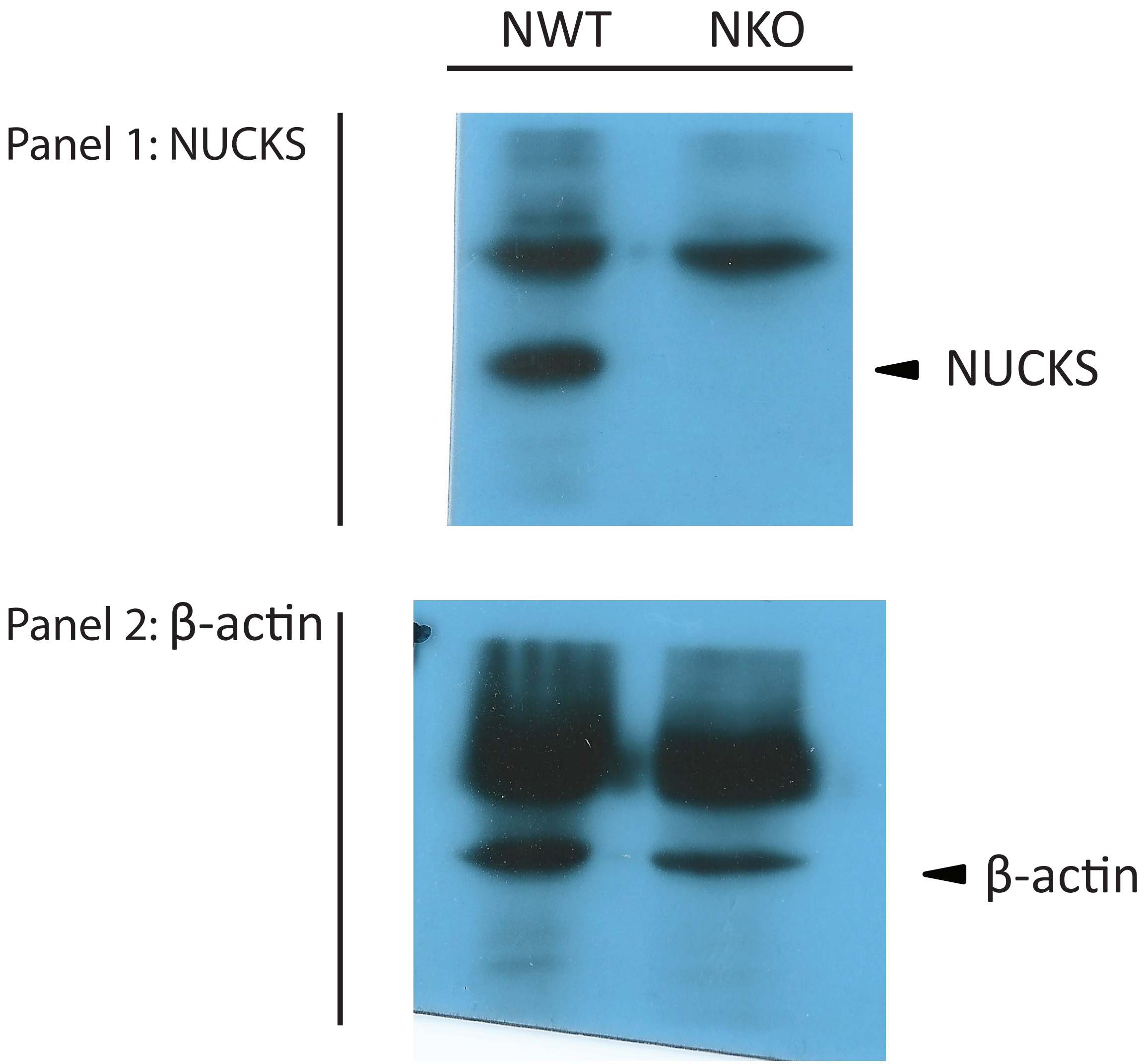

(Full-length gel image)

Supplementary Figure- S2

A **i**

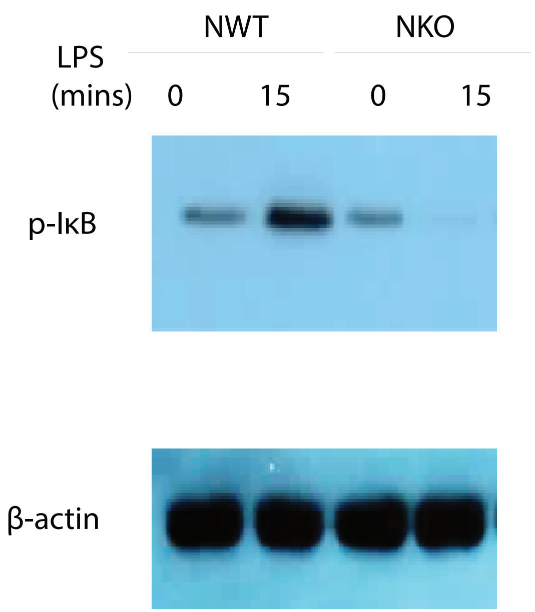

B **i**

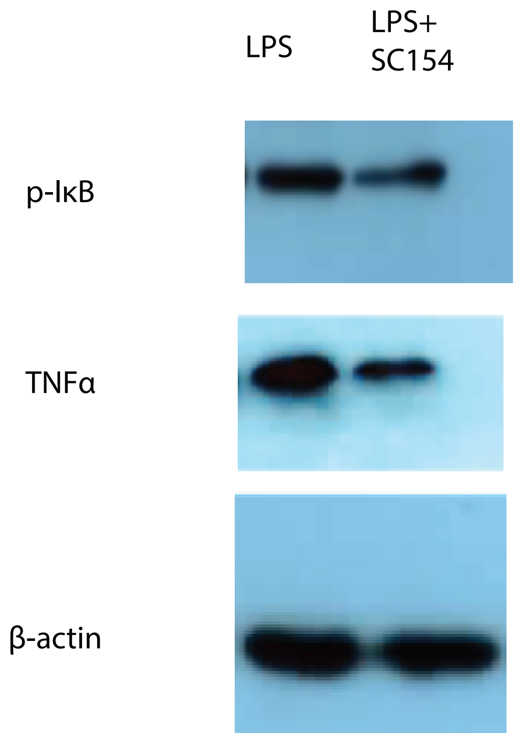

(Full-length gel image)

Supplementary Table 1: Primers for Real-time RT PCR.

		Primers	
**Transcripts**	**Accession Number**	**Forward Primer 5'->3'**	**Reverse Primer 5'->3'**
**Immflammatory Cytokines**			
mIL1A	NM010554.4	GAC AGT ATC AGC AAC GTC AA	TCA CTC TGG TAG GTG TAA GG
mIL1B	NM008361.3	GAG TGT GGA TCC CAA GCA AT	TAC CAG TTG GGG AAC TCT GC
**Angiogenesis Cytokines**			
mVEGF	NM_001025250.3,NM_009505.4, NM_001025257.3	CCC GAC GAG ATA GAG TAC AT	CTG GCT TTG GTG AGG TTT
mPEDF	NM011340.3	CCC GAC TTC AGC AAG ATT AC	CCC TCA GAA CAA AGA GGA AAG
**House Keeping**			
GAPDH	NM008084.2	CCA CTC ACG GCA AAT TCA	AGT AGA CTC CAC GAC ATA CTC

Keys: m=mouse

Figure Legends:

Supplementary Figure 1. Confirmation of Genotypes of Mice for NWT and NKO by Western Blot Analysis. Specific antibody for NUCKS (Abcam 1:100) was used to confirm the genotypes.

Supplementary Figure 2. Full Gel Images of Western Blot Analysis of Figure 8A and 8B.

Supplementary Table 1. Details of the Specific Primers for Various Cytokines for Real Time –RT-PCR Analysis. This table shows the sequences and specific information for the sense and anti-sense primers for inflammatory cytokines (IL1A and IL1B), angiogenic and antiangogenic cytokines (VEGF & PEDF, respectively) and the house-keeping gene (GAPDH).
